# Aging Successfully: Possible in Principle? Possible for all? Desirable for all?

**DOI:** 10.1007/s12124-020-09513-8

**Published:** 2020-01-27

**Authors:** Hans-Werner Wahl

**Affiliations:** Network Aging Research & Institute of Psychology, University of Heidelberg, Bergheimer Straße 20, 69115 Heidelberg, Germany

**Keywords:** Successful aging, Aging well, Well-being, Physical functioning, Adaptation

## Abstract

The human lifespan is constantly increasing across the world. Therefore, the question whether aging can take a “successful” route likely never has been as up-to-date as today. Still, gerontology continues to struggle with the concept of successful aging. In part I of this work, I outline six models of successful aging currently most discussed in aging science. Next, I compare the models according to four indicator domains: Psychologically oriented, socially oriented, bio-physical functioning oriented, and other. In part II, I address three key questions related to successful aging: Is successful aging possible in principle? Is it possible for all? Is it desirable for all? Regarding the first question, the conclusion based on empirical data is that across all models except the biological model aging successfully is possible in principle. Hence, I answer this question with a “YES, BUT.” Successful aging at the bio-level so far seems very limited. In terms of question 2, expecting largely increasing portions of older adults fulfilling various of the so far established criteria of aging successfully in the future seem overly optimistic. Hence, I answer this question with “NO.” For question 3, I critique the at first glance obvious persuasiveness of successful aging as a generally to be achieved end state. For example, it may be that norms of successful aging undermine old age’s cultural richness in the future. Hence, I answer this question with “NOT SURE.”

There can be no doubt that we currently see a ‘new aging.’ One may argue that this has always been the case in that aging certainly was different in antique Rome as compared to the times of hunters and collectors and certainly also in the time of enlightenment as compared to medieval age. However, it seems fair to state that something qualitatively different happened to human aging since the end of the nineteenth century particularly in what many call the developed countries.

First, life expectancy at birth has increased within about 120 years in unprecedented dynamic in the developed countries. For example, in Germany life expectancy at birth showed an increase since the end of the nineteenth century from about 46 years to about 80 years at present, averaged across gender (Our World in Data 2019). This increase is not too far from doubling in somewhat more than a century, it is about a tripling compared to the estimated the situation in Ancient Greece and Rome with a life expectance of 20 to 35 years and about a quadrupling compared to the estimated situation 300,000 years ago, when human kind emerged on the planet (Finch 2010). Yet, as demographers tell us based on sound population-based calculation of birth and death population-based data, this is not the end and even not the beginning of the end. For example, average life expectancy will ‘easily take’ the 80 years margin in many developed countries in the years to come; it already surpassed the age of 85 years in female Japanese and the age of 80 years in Irish males (Salomon et al. 2012; Our World in Data 2019). Still, such average life expectancy is far from what seems possible in principle. The so far best documented case is Jeanne Calment, who lived in Southern France and died in 1997 at the age of 122.4 years.^1^ Hence, the plasticity of what we have in terms of ‘reserve capacity in life expectancy’ seems like a large still ‘unlived’ but in principle ‘possible to live’ reservoir.

Second, although the demographic situation in the so-called developing countries is still not comparable to the one in the developed world, fast demographic aging has also gained a major role in developing or threshold countries. For example, according to Salomon et al. (2012), life expectancy at birth in Senegal in 1990 was 56.8 years in men and 60.9 years in women; in 2010, it has raised to 63.5 years (men) and 67.1 years (women). Similarly, life expectancy at birth in Egypt in 1990 amounted to 62.4 years in men and 67.0 years in women; in 2010, men’s life expectancy has climbed to 68.0 years in men and 73.4 years in women. More rarely, opposite dynamics can be observed. For instance, at the world’s likely lowest level life expectancy at birth for men decreased in Haiti from 53.3 years in 1990 to 32.5 years in 2010. By and large, human kind has been (and likely continues to be) very successful in extending its average lifespan across the world, although some negative examples also exist.

Third, certainly a number of factors are important for differences in life expectancies among countries. Among the major influences are differences in the survival of children, socio-economic disparities, and, as has been recently shown, differences in premature death occurrences with countries lower in premature death generally revealing higher life expectancy rates (Vaupel et al. 2018).

Yet, would reducing the term ‘successful aging’ to mere life extension be convincing? The Gerontological Society of America (GSA) founded in 1945 already in the 1950’s rightly advertised the ambition to ‘add life to years and not just years to life.’ This claim has gained much actuality due to what we currently see in terms of increase in ‘healthy life expectancy’ (Harper 2014), rather positive cohort effects comparing older adults of today with those 20 or 30 years ago in a range of outcomes (Drewelies et al. 2019), and what is frequently called the ‘third age’ as a new and quite proactive period in the late part of the human lifespan (Dittmann-Kohli and Jopp 2007; Laslett 1989). In stark contrast, the ‘fourth age’ following the third age has been characterized as a life period of pronounced multi-morbidity, physical and mental frailty, and dependency (Baltes and Smith 2003; Laslett 1989). In a sense, the third age may be qualified as the most welcomed, whereas the fourth age may be seen as the most disliked part of the lifespan in post-modern life (see also Gilleard and Higgs 2010, 2011).

Closing this introduction, I would like to underscore that all of what I do in the paper is enriched by the spirit of Dieter Ferring, who passed away unexpectedly in the summer of 2017. In particular, his ambition to strive for sustainable combinations of pure research and evidence-based efforts toward applied research and serving older adults’ quality of life have been very inspiring for me while treating the issue of successful aging. Dieter also discussed in one of his posthumously published papers the issue of successful aging (Ferring 2018). I will come back to his skeptical view of the concept of ‘successful’ applied to aging in the closing section of this work.

## Part I: Establishing the Idea of Aging Successfully in Aging Science

We may start by asking why the term successful aging currently elicits far more than 100 million entries in *Google* (search on November 1, 2019). Interestingly, the concept is not that new in aging science and already appeared in writings of key figures in early gerontology and developmental science such as Pressey and Simcoe (1950), Havighurst and Albrecht (1953), and Havighurst (1961). Interestingly, the concept of ‘satisfaction with life’ the measurement of which was for the first time addressed in the article by Neugarten et al. (1961) also found a first major treatment in the same *Volume 1* issue of the journal *The Gerontologist* in which Havighurst’s (1961) work also appeared. Attempts to organize existing models of successful aging have been manifold (e.g., Tesch-Römer in press) and may be critiqued, for instance in terms of missing clear borderlines. Therefore, the following categorization should be seen as a heuristic approach that includes overlap and does not exclude important similarities among the models.

### Model 1: Successful Aging as Maintaining Subjective Well-Being while Aging

Following this early seminal work by Havighurst and Neugarten, the major answer of gerontologists to what successful aging means has been a high level of life satisfaction, or, broader framed, of well-being related indicators. There are various conceptions of subjective well-being. A well-established differentiation is the one between a cognitive and an affective component, which divides well-being into (cognitive) judgments about a person’s satisfaction with life (and life-domains) and affective well-being, echoed in positive and negative affect (Kunzmann et al. 2000). The overall answer of this approach has been that successful aging means an as high as possible well-being across later life until death. The approach strongly relies on research supporting the *paradox of subjective well-being*, hence the well-documented phenomenon that the level of psychological well-being remains rather stable across old age despite the many loss experiences associated with getting older, in particular health-related losses (Kunzmann et al. 2000).

Additionally, subjective evaluations able to speak to successful aging are reflected in the construct of subjective health, which has been shown to develop different from objective health conditions; for example, whereas objective loss in functional capacity and number of simultaneously existing diagnoses clearly increase with aging, subjective evaluations of health remain rather stable (e.g., Wettstein et al. 2016).

Besides the psychological and health-related domain, subjective evaluations also address the social domain as a major area. In particular, if aging people rate their overall social situation as being low in terms of loneliness and high in social integration, then this may qualify the situation as successful aging (Faber 2001).

### Model 2: Successful Aging as Achievement of Objective Criteria

Rowe and Kahn (1987) set out in their much cited article in *Science* a model of successful aging that has been highly influential ever since (Rowe and Kahn 1997, 2015). A major driving force behind Rowe and Kahn’s approach has been that aging science struggled long to leave a deficit view on aging behind. Therefore, Rowe and Kahn (1987) strived to identify in their article in *Science* rather objective and objectively measurable criteria for successful aging, not subjective interpretations and evaluations of aging processes and outcomes. Rowe and Kahn’s definition contains as a first key element three components echoing three criteria for qualifying aging as successful: (1) low probability of illness and related disability, (2) high level of cognitive and physical functioning, and (3) active engagement with life. Rowe and Kahn claimed that achieving these three criteria as much as possible allows for the best life possible in the late period of the lifespan. A second key element of Rowe and Kahn’s approach has been their argument that ‘usual aging’ not reaching full achievement of the three criteria is currently dominating the realities of aging; however, they also expect but so far unused potentials of aging well will unfold in the future, when life-long preventive efforts come to full exploitation. That is, Rowe and Kahn’s model comes with a strong visionary component of what might be possible in later life and thus in a sense with a utopian view of aging that can be seen only in a minority of currently aging individuals.

### Model 3: Successful Aging as Fulfilment of Fundamental Norms/Values of a Good Life

This idea of successful aging is driven by the assumption that qualifying individuals as successfully aging requires reference to normatively framed ideal states or value judgments on the “good life.”. First, a classic example for this position is the theory of life-long personality development by Erikson 1950; see also Erikson and Erikson 1997), according to which ego-integrity (in the third age) and gero-transcendence (in the fourth age, relying on a concept proposed by Tornstam 1989) are the ultimate goals of late human development. Second, the concept eudaimonic well-being (also called psychological well-being) contains according to Ryff’s (1989) multidimensional model of psychological well-being six dimensions for which older adults are striving for in order to age successfully. The six dimensions include self-acceptance, autonomy, personal growth, purpose in life, environment mastery, and positive relationships. Third, maintaining health as long as possible is major ideal state that is reflected in the construct of “healthy aging” (World Health Organzation 2019) as well as in the long-time established construct of health-related quality of life (Center for Disease Control and Prevention 2019).

### Model 4: Successful Aging as Using Efficient Adaptational Strategies

Behind this idea of successful aging is the proposition that researchers in the area should avoid as much as possible subjectively rated, objectively assessed, or ideal states. The most established approach is the model of *selective optimization with compensation* (SOC) introduced by Baltes and Baltes 1990; see also Freund et al. 2017). The model states that three core processes need to be coordinated throughout life in order to achieve the best possible gain-loss balance and optimal adaptation. Selective processes are necessary because humans need to make decisions throughout the lifespan where and how to invest their efforts. Compensatory processes also play a role across the lifespan because of the possibility of failure and loss of action at any point in life that needs to be counter-balanced. Optimization requires the reciprocal and dynamic interplay of selection and compensation and addresses the possibility of developmental growth throughout life—that is, growth opportunities in one behavioral domain may be optimized by selecting out of another, perhaps less loss-affected domain. Although the SOC model emerged out of psychological aging research, it certainly reveals cross-over implications to the social domain (Baltes and Carstensen 1999) and the physical functioning domain (Gignac et al. 2000). In fact, socio-emotional selectivity theory (e.g., Carstensen 2006) posits that older adults experience an increasingly constrained future time perspective, which “forces” them to invest into the intimacy of positively valued social relations. Another process helping to age successfully may be seen in research on subjective aging which showed that feeling younger than one’s chronological age is adaptive and serves on the longer run one’s health and well-being (Westerhof et al. 2014).

### Model 5: Successful Aging as What Older Adults Themselves Value as Important for their Life

Studying laypersons’ perspectives underline that the understanding of successful aging by older adults themselves is facet-rich (Bowling 2006). Jopp et al. (2015), for example, found that, compared to existing psychological theories, laypersons’ view on successful aging indeed reveals more subdimensions, encompassing health, activities, social relations, finances and psychological resources (such as well-being), and attitudes and life-management skills.

### Model 6: Successful Aging as Slowing or Abandoning Biological Aging

The major argument here is that, compared to all (more or less efficient) medical approaches to counteract disease in later life, it may be more effective to go more general and change the biological aging process per se in terms of reducing the speed of aging and prolong the healthy lifespan. A range of efforts are currently underway in biogerontological research to approach this goal such as intake of Metformin, caloric reduction, or transmitting blood of younger organisms in mice to older mice organisms (e.g., Barzilai et al. 2016).

### Juxtaposing Models of Successful Aging

Taking all models of successful aging intro consideration, Table 1 presents a comparative view. Doing so, a distinction is made between the nature of indicator used for qualifying successful aging (psychologically oriented, socially oriented, bio-physical function oriented, other) and the six approaches of successful aging described before. Table 1 allows for some observations. For one, all approaches except the biogerontological model strive to address all three sets of indicators able to qualify successful aging. Second, the fundamental life goal / ideal state model seems to come with the most extensive list of (psychological) indicators, which can be seen as an advantage (high differentiation) or disadvantage (heterogeneous compilation of ideal states, which easily can be extended further). Third, older people’s view of successful aging seems not that different from “scientific views”, but tends to be more extensive overall; it is also the only approach, which also mentions financial resources. Fourth, the adaptational strategies approach with its major content of the SOC model may be seem as the most parsimonious conceptualization, because it fully relies on the orchestration of three omnibus processes that operate in different life domains. Fifth, the social and health domains are addressed by all approaches except the biogerontological approach. Finally, the psychological indicators domain appears as particularly diverse in what kind of constructs are considered.

**Table 1:** Comparative View of Successful Aging Approaches

Indicators Used for Successful Aging (Key Examples)	Model 1: Successful Aging as Maintaining Subjective Well-being While Aging	Model 2: Successful Aging as Achievement of Objective Criteria	Model 3: Successful Aging as Fulfilment of Fundamental Norms/Values of a Good Life	Model 4: Successful Aging as Using Efficient Adaptational Strategies	Model 5 Successful Aging as What Older Adults Value as Important for Their Life	Model 6: Successful Aging as Slowing or Abandoning Biological Aging
Psychologically Oriented Indicators	High Well-being (Cognitive and Affective)	High Cognitive Functioning	Ego-Integrity, Gero Transcendence, High Self-acceptance, High Autonomy, High Environmental Mastery, High Personal Growth, High Purpose in Life,	Selective Optimization with Compensation (at the Psychological Level)	Positive Views on Life and Aging, High Well-being, High Life-Management Skills	–
Socially Oriented Indicators	High Satisfaction with Social Relations, Low Loneliness	High Social Engagement	Positive Social Relationships	Selective Optimization with Compensation (at the Social Level)	Highly Valued Social Relations	–
Bio-Physical Functioning Oriented Indicators	High Subjective Health, High Subjective Rating of Physical Functioning	Low Disease Occurrence / High Functional Ability	Highest Health Status Possible	Selective Optimization with Compensation (at the Physical Function Level)	Optimal Health, High Physical Functioning	Longer Lifespan, Slowed-down Biological Aging Processes, Reduced Disease Rate
Other Indicators	–	–	–	–	Good Financial Situation	–

## Part II: Treating Three Questions on Successful Aging

Against what has been said above, three questions will be treated in this second section of the paper: (1) Is successful aging possible in principle? (2) Is it possible for all? (3) Is it desirable for all? The first question addresses the current potential of aging as it emerges from established findings of aging science. The fundamental issue is whether aging as it currently appears can at least in principle follow a course that we may qualify as “successful” in the various senses of “successful aging” distinguished above. Hence, this would be the proof of principle issue. The second question targets constraining conditions that may promote in some subgroups or undermine in other subgroups or even in the majority of older adults the unfolding of their reserve capacity to achieve successful aging, although such capacity is available in principle. Regarding the third question, I take a still more normative stance in that I ask whether it would be indeed a great vision, if all older adults would uniformly strive for the goal of successful aging. As a disclaimer, word limitation does not allow to consider the full content as depicted in Table 1 for discussing the three question: I have to be selective but do hope that general tendencies will nevertheless become sufficiently clear.

### Is Successful Aging Possible in Principle?

A quick journey across models of successful reveals the following, when it comes to existing empirical data able to speak to these models^2^:

#### Successful Aging as Maintaining Subjective Well-Being while Aging

Focusing a selection of the key indicators of this approach at the psychological indicator level, cross-sectional and longitudinal research on mean-level change in overall affective well-being suggests high stability of *positive affect* across the adult lifespan, with only some age-related decrease in old and very old age (e.g., Charles et al. 2001). With respect to *negative affect*, longitudinal (Charles et al. 2001) as well as cross-sectional data (e.g., Carstensen et al. 2000) have provided evidence for its *decline* with advancing age. As Kunzmann et al. (2000) showed with data from the Berlin Aging Study (BASE), a higher age was associated with a lower level of negative affect. Further, BASE reported that older adults in the age range from 70 to 95 years showed, as expected, significant declines with regard to vision, hearing, mobility, social participation, and their ability to live independently at home. Yet, subjective well-being only showed minor decline across this age range (Smith and Baltes 1999; see also Wahl and Schilling 2018).

Regarding the level of socially oriented indicators, a recent meta-analysis by Mund et al. (2019) summarized the findings on loneliness from 75 *longitudinal* studies with at least 1-year observational intervals and a broad age range (from 6 to 80+ years of age). Mund et al. (2019) found rather high mean-level stability in loneliness across the lifespan and no significant age effect.

At the level of bio-physical functioning indicators, subjective health has repeatedly been found to show only limited decline as people age, although the number of illnesses and, technically, of diagnoses increase with age. For example, Wettstein et al. (2016) found in a sample of advanced old age participants aged 80 years and older that objective indicators of physical functioning such as reflected in gait capability revealed a clear decline across an observational period of 8 years, whereas the subjective rating of health remained rather stable.

#### Successful Aging as Achievement of Objective Criteria

This model focuses at the psychological indicators level on cognitive functioning. In this area, two findings are of particular relevance. For one, data from the Seattle Longitudinal Study (Schaie 2013) across 28 years of observation in cognitive components, such as inductive reasoning and numerical operations decline between the age range between 25 and 88 years in the magnitude of 1 SD. Another view on the same indicators shows that there is a great deal of stability until at least the age band of 60 to 70 years. Second, a number of existing data confirm that relatively high cognitive functioning can be observed until death, hence even in those above the age of 90 years (Lindenberger and Reischies 1999).

With respect to socially oriented indicators, we already addressed the issue of loneliness. In addition, Wrzus et al. (2013) showed in their meta-analysis that network size particularly in intimate and family related relations remains pretty constant until advanced old age. This is also consistent with the empirically well-established predictions of socio-emotional selectivity theory (Carstensen 2006), hence aging individuals invest much in intimate relations and thus proactively maintain personally meaningful social relations.

At the level of bio-physical indicators, the age-related challenge may be seen as most pronounced. Still, epidemiological data reveal that even in those over the age of 90 years, about 29% of those between 90 and 94 years old, 11% in in 95–99 year olds, and 8% of centenarians report having *no difficulty* in conducting activities of daily living (Berlau et al. 2009).

#### Successful Aging as Fulfilment of Fundamental Norms/Values of a Good Life

In terms of psychological indicators, Ryff and Keyes (1995) found that personal growth and purpose in life were lowest in older adults compared to individuals in young adulthood or midlife. In contrast, positive relations, environmental mastery, self-acceptance, and autonomy were either highest in old age or remained rather stable. Hence, what we see here is a picture of pronounced multidirectionality.

#### Successful Aging as Using Efficient Adaptation Strategies

In terms of lifespan trajectories of SOC processes, an increase from young adulthood to old age has only been observed in selection (Baltes et al. 2006). Selection seems to become particularly important in old age due to resource loss. In addition, both optimization and compensation decrease from midlife to old age. However, available data support that the successful orchestration of SOC processes in principal works well until death (Freund and Baltes 1998), although efficient SOC able to balance gains and losses becomes quite a difficult endeavor in advanced old age (Baltes 1997).

#### Successful Aging as What Older Adults Value Themselves as Important for their Life

An important finding speaking to this model is that up to three quarters of older adults have rated themselves as aging successfully in a British survey study with those 50 years and older (Bowling and Dieppe 2005).

#### Successful Aging as Slowing or Abandoning Biological Aging

This idea of successful aging still has mostly remained at an experimental stage so far. Although there are encouraging findings based on animal models (e.g., that caloric reduction goes along with prolongation of the lifespan in the worm *C. elegans*), generalization to humans still is questionable or under controversial debate (e.g., De Cabo et al. 2014; Kirkwood 2017).

#### Conclusion

I conclude that across all models except for the biological model successful aging in aging humans seems possible in principle based on empirical data. Though, some differences do emerge in that the likelihood for aging successfully seems the highest in the area of psychologically oriented indicators. Differentiation is nevertheless also in need at this level, given the empirically found decreasing rates in life goals such as personal growth and purpose in life. Similarly, taken the level of socially oriented indicators, the likelihood of aging successfully seems considerable until the end of life. The major challenge lies at the bio-physical oriented level in that rates of high scorers are increasingly low as people move into the Fourth age. However, individuals without serious functional impairment can also be found in those 90 years and older and thus successful aging seems possible also in advanced old age. Taken all together and although taking some needed differentiation into account, the answer on question 1 would be a “YES, BUT.” Successful aging at the fundamental bio-level so far seems hard to achieve.

### Is Successful Aging Possible for all?

Overall, searching for answers on this question is a complex endeavor and will likely end in an ongoing debate. Still, I believe that a preliminary answer based on currently available evidence can be given. Notably, Rowe and Kahn more recently made a call for “Successful Aging 2.0” (Rowe and Kahn 2015). As they now argue, as societies continue to grow older, aging research should analyze policies that allow societies to deal successfully with the benefits and risks of demographic change, i.e. policies that foster productivity, cohesion, resilience, and sustainability in aging societies, and that “facilitate successful aging at the level of the individual” (Rowe and Kahn 2015, p. 2). Hence, responsibility for achieving successful aging is seen as a complex intertwining of political, societal, and individual efforts. Yet, from what situation are we starting to be optimistic that we all have a considerable chance to age successfully? In the following, I will examine three sets of empirical data to provide an answer: (1) Rates of successful aging; (2) ongoing dynamics between healthy and unhealthy life years as a future reality of aging; and (3) recent research on the fourth age and terminal decline in various areas of functioning. Due to space constrictions, I will not consider all models of successful aging equally, but concentrate on the recently most debated one, i.e., the Rowe-Kahn approach.

#### Rates of Aging Successfully

Optimism that we will all achieve successful aging would need an empirical baseline across a range of cultures / countries that would nurture confidence that the goal is realistic at least in the next 20 to 30 years to come. When we refer to the Rowe and Kahn’s (1997) model combining the three objective indicators of high cognitive functioning, low disease level, and high social engagement, Hank (2011) arrived based on data from the SHARE study that older adults in Denmark reveal a rate of 21.1%, whereas the percentage in Polish older adults would be only 1.6%. McLaughlin et al. (2010) conducted a similar analysis based on date of the Health and Retirement Study (HRS), one of the most robust survey data sets worldwide. They found a rate of successfully aging adults in the 75–84 years old of 6.5% and those over 85 years of 1.7% applying Rowe and Kahn’s criteria. When the window of criteria is flexibly opened and different models of successful aging (e.g., those relying on objective and subjective indicators) are taken into account as Cosco et al. (2014) did in their analysis, the rate increases to 26.0%.

The picture looks far rosy when we only rely on subjective evaluations of life models. For example, in data provided by Wahl and Schilling (2018) in older adults beyond the age of 80 years, using a 11-point life satisfaction rating between 0 (“low”) and 10 (“high”), about 50% would be positioned above a threshold of 8 points. In the already cited Bowling and Dieppe’s (2005) study of older individuals from the UK, 75% rated themselves as aging successfully.

#### Healthy and Unhealthy Life Expectancy

In an international study comparing a large selection of 187 countries and using disability as health indicator, evidence was found for expansion of disability in late life (Salomon et al. 2012). Between 1990 and 2010, worldwide total life expectancy increased faster than healthy life expectancy, with each 1-year increase in life expectancy at birth associated with a 0.8-year increase in healthy life expectancy. In addition, although there were expectable differences between countries, an overall trend of disability expansion was observed in the clear majority of countries. For instance, in the U.S., life expectancy in poor health increased for women from 10.5 years in 1990 to 11.0 years in 2010; for men the increase was even larger (from 8.7 years in 1990 to 9.7 years in 2010; Salomon et al. 2012, p. 2152). Hence, while healthy life expectancy is further increasing over time (Harper 2014), Salomon et al.’s data suggest that years gained in good health will be accompanied by additional years in poor health. It seems quite unlikely that this trend found in data comparing the years 1990 and 2010 has substantially changed meanwhile.

#### The Fourth Age and Terminal Decline as a Challenge for Successful Aging

The globally valid pattern on stability in many psychological indicators particularly in subjective evaluations of life changes often dramatically as people move into the Fourth Age and the last years of life. For example, Vogel et al. (2013) found with data from the Longitudinal Aging Study Amsterdam (LASA) that time-to-death accounts for more intraindividual variance and reveals a better fit to the data than chronological age, indicating terminal drop of affective well-being in terms of decreases of positive affect and increases of negative affect (see also Gerstorf et al. 2008, 2010). When resources such as functional capacity, sensory functioning, and cognitive abilities become increasingly compromised in the Fourth Age and in the terminal phase of life, it seems to become very difficult for many individuals to fulfill the criteria of most models of successful aging considered above (Baltes and Smith 2003).

#### Conclusion

Visions that much increasing portions of older adults will fulfill the criteria of aging successfully in the future seem overly optimistic, if not wishful thinking. Rates of successful aging in Rowe and Kahn’s sense are very low particularly in those above the age of 85 years, but even in those 74 to 84 years of age and it seems unrealistic that such rates will for instance triple in the next 20 to 30 years to come. And even if so, this would mean for example that only about a fifth of those in the age band of 74 to 84 years and only about every 12th out of 100 of those above the age of 85 years would age successfully. The situation is even worst, when unhealthy life expectancy happening in the very last part of the lifespan and thus in the fourth age is further increasing, which can be expected. Finally, even the models of successful aging relying on subjective evaluations of one’s life, which arrive on average at more positive views come under considerable pressure in the terminal life phase. Taken all together and although taking some needed differentiation into account, the answer on question 2 would be a “NO”, even when we exclude the biologically driven model of successful aging.

### Is Successful Aging Desirable for All?

Obviously, I enter with this question the arena of justification of norms of “successful aging.” As a psychologist, I will remain cautious here, knowing that empirical data have nothing to do with normative decisions (naturalistic fallacy). Still, I believe that some treatment of normative issues is in place, because the term “success” seem to strongly suggest that such striving “must” be good for every older individual and what philosophers might call a prudence rule (in German: Klugheitsregel) for aging populations at large. I see at least three issue helpful to consider and critique such a recommendation: (1) A general demand for successful aging may put new pressure on aging individuals; (2) A general demand for successful aging may undermine the unfolding of the cultural richness of aging in the future; and (3) A general demand for successful aging will get into constant conflict with the “low rate problem” of successful aging, particularly when it comes to models of successful aging relying on objective indicators.

#### General Demand for Successful Aging May Put New Pressure on Aging Individuals

Can we imagine a person who does not intend to age successfully? At first glance, such a person seems hard to imagine. However, what happens if there are aging individuals telling us as researchers that “successful” is not a category they like to impose on their later course of life as a meaningful category? (see also Bowling and Dieppe 2005). At least, as it seems, aging science is on its way with the term successful aging to offer (if not impose) a norm on good aging to older adults and society at large which may make it hard to distance or escape from. If successful aging will become the new norm of aging individuals in the future in terms of what they should do and feel, what some have called the “new freedom of aging” (Rosenmayr 1983), particularly the potential of the third age as a life period with many opportunities (see again Laslett 1989), may get lost again. In addition, an emerging successful aging imperative may increasingly force aging individuals to engage as much as possible in maintaining “youthfulness” and “agelessness” as long as possible. Following such goals may indeed aggravate the needed awareness of and adjustment to age-related change and thus come with more cost than benefit for older adults in the longer run.

#### General Demand for Successful Aging May Undermine the Unfolding of the Cultural Richness of Aging in the Future

Most aging scientist are seeing the pronounced heterogeneity of aging as kind of a “virtue” of the last phase of life (e.g., Maddox 1987). Aging scientists are not getting tired in making the claim that there is not such a thing like “old age” or “aging” and that recognizing diversity is possibly among the most crucial evidences generated so far by gerontological research. Given the diversity in late human life styles, preferences, and meaning making (e.g., Nelson and Dannefer 1992), it may be more constraining than enriching, if ‘conformity’ in achieving and maintaining the highest function, well-being, and autonomy would become a the culturally anchored goal for complete older populations. In fact, a social norm of aging successfully may undermine the cultural richness and colorfulness of aging as it will unfold in the future. Though this argument may apply differently to different models of successful aging. In particular, a major strength of the SOC model is seen in its relativity, hence it is open for very different ways and means to live a good life and thus, as Baltes (1997) noted, flexible enough for infinite variations of phenotypic realizations. However, I am not convinced, Baltes (1997) argued, that the strong associations of the SOC model with economic success and productivity are positively overshadowed by its broad array of applicability and parsimony finally full holds. Indeed, the application of SOC as the sole prudence rule of development might undermine creativity, curiosity, tolerance for ambiguity, and searching for the unexpected and unknown in life.

#### General Demand for Successful Aging May Continue to Get into Conflict with the “Low Rate Problem” of Successful Aging

Assuming that considerable subgroups of future older adults will face much difficulty to fulfill at least some of the criteria of the models of successful aging as previously discussed, it may become of critical importance to avoid the stigma of “unsuccessful aging.” Therefore, Tesch-Römer and Wahl (2017) argue that successful aging with significant functional impairment and severe care needs will not result in uselessness of the term. As they argue, socio-emotional and communion-like elements inherent in high-level informal or professional care have the potential to enable successful aging even in situations of pronounced vulnerability such as in advanced old age. Importantly, this would not mean that now all old and very old adults would be aging successfully. Although long-term care has changed over the last decades, and a variety of innovations have transformed the field (Gaugler 2015), there is no doubt that quality of care differs between and within settings. Hence, the reality of long-term care also is in need of criteria of successful aging. The hope is indeed that an extended and more flexible concept of successful aging able to also apply to aging individuals with severe care needs might help to change the organization and practice of long-term care and contribute to positive changes in the culture of care in long-term care settings (see also Boll et al. 2018). In a sense then, what Tesch-Römer and Wahl 2017; see also Wahl and Tesch-Römer 2018) recommend is to take action to transcend the traditional “More is Better” connotation inherent in many concepts of successful aging. Instead, creating states of socio-emotional and care-driven richness in life situations for which concepts such as optimization no longer apply may be a way out of the “low rate problem.”

#### Conclusion

At first glance, it seems obvious that aging successfully is a desired goal for all. Further, if biogerontological progress will be successful in the longer run in terms of slowing aging and reducing the late-life disease burden, the idea of aging successfully may see widespread dissemination and acceptance in aging populations, but also in younger ages. In the more likely scenario of increasing numbers of frail older adults in the future (Salomon et al. 2012), however, I see a major need to integrate models of care into models of successful aging to avoid the stigmatization of “aging unsuccessfully” for a considerable subportion of the aging population. Taken all together and although considering some needed differentiation, the answer on question 3 would be “NOT SURE.” Being more flexible in what successful aging might mean may reduce some of the normative pressure of a narrow successful aging definition and thus allow pluralism as a leitmotiv for future successful research and practice.

## Resume

Treating issues of successful aging evokes a number of dilemmas and it is therefore surprising that the concept has gained so much prominence and citation rates. In particular, existing models able to conceptually underfeed and consult older adults’ striving to age well are quite diverse and in part contradictory. In addition, aging as process and outcome is certainly not fixed. In fact, research on the effects of historical change among older adults in developed nations such as the US, Sweden, and Germany suggests by and large a success story. For example, data from Germany indicates that 75-year old Berliners today are cognitively much fitter than the 75-year-olds of 20 years ago (e.g., Gerstorf et al. 2015; see also Drewelies et al. 2019). At the same time, challenges connected with advanced old age and a likely extension of the period of unhealthy years dampen the positive messages converted by such cohort findings (Tesch-Römer and Wahl 2017). However, I fully concord with Baltes’s view that “States of deficit and limitations, such as the fourth age, are powerful catalysts for scientific and cultural innovation.” (p. 377).

In closing, Dieter Ferring (2018) certainly has been right, when he critiqued a too individualistic position of most models of successful aging thus neglecting to a large extent the role of context. I fully accord with Dieter that more conceptual and empirical investment in explicating the co-constructing role of context from the micro to macro and cultural value systems is needed in future research on successful aging (see also Valsiner 1994; Wahl and Gerstorf 2018). Indeed, continuing the debate on successful aging in engaged ways might be more fruitful for future gerontology than expecting definite outcomes or consensus.

## Footnotes

1 Recently, doubts were raised that not Jeanne Calment but her daughter lived until 1997. However, acknowledged demographers such as Prof. Jean-Marie Robine, Montpellier, have meanwhile carefully double-checked and argue that all relevant information on J. Calment is correct (Robine et al. 2019).

2 Even more than what can be empirically observed might be possible in terms of successful aging in the future. However, I am following here the logic that if one case of successful aging according to certain criteria can be observed, then such a variant of successful aging is possible in principle.

## References

[CR1] Baltes PB (1997). On the incomplete architecture of human ontogeny: Selection, optimization, and compensation as foundation of developmental theory. American Psychologist.

[CR2] Baltes PB, Baltes MM, Baltes PB, Baltes MM (1990). Psychological perspectives on successful aging: The model of selective optimization with compensation. Successful aging: Perspectives from the behavioral sciences.

[CR3] Baltes MM, Carstensen LL, Bengtson VL, Schaie KW (1999). Social-psychological theories and their applications to aging: From individual to collective. Handbook of theories of aging.

[CR4] Baltes PB, Smith J (2003). New frontiers in the future of aging: From successful aging of the young old to the dilemmas of the fourth age. Gerontology.

[CR5] Baltes, P. B., Lindenberger, U., & Staudinger, U. M. (2006). Life-span theory in developmental psychology. In W. Damon & R. M. Lerner (Eds.), *Handbook of child psychology: Vol. 1. Theoretical models of human development* (6th ed., pp. 569-664). New York, NY: Wiley.

[CR6] Barzilai N, Crandall JP, Kritchevsky SB, Espeland MA (2016). Metformin as a tool to target aging. Cell Metabolism.

[CR7] Berlau DJ, Corrada MM, Kawas C (2009). The prevalence of disability in the oldest-old is high and continues to increase with age: Findings from *The 90+ Study*. International Journal of Geriatric Psychiatry.

[CR8] Boll, T., Ferring, D., & Valsiner, J. (Eds.). (2018). *Cultures of care in aging*. Information Age Publishing.

[CR9] Bowling A (2006). Lay perceptions of successful ageing: Findings from a national survey of middle aged and older adults in Britain. European Journal of Ageing.

[CR10] Bowling A, Dieppe P (2005). What is successful ageing and who should define it?. BMJ.

[CR11] Carstensen LL (2006). The influence of a sense of time on human development. Science.

[CR12] Carstensen LL, Pasupathi M, Mayr U, Nesselroade JR (2000). Emotional experience in everyday life across the adult life span. Journal of Personality and Social Psychology.

[CR13] Center for Disease Control and Prevention (2019). Health-related quality of life. https://www.cdc.gov/hrqol/index.htm. Download on November 4, 2019.

[CR14] Charles ST, Reynolds CA, Gatz M (2001). Age-related differences and change in positive and negative affect over 23 years. Journal of Personality and Social Psychology.

[CR15] Cosco TDA, Prina AM, Perales J, Stephan BCM, Brayne C (2014). Operational definitions of successful aging: A systematic review. International Psychogeriatrics.

[CR16] De Cabo R, Carmona-Guitierrez D, Bernier M, Madea F (2014). The search for antiaging interventions: From elixirs to fasting regimens. Cell.

[CR17] Dittmann-Kohli F, Jopp D, Bond J, Dittmann-Kohli F, Peace SM, Westerhof G (2007). Identity, self, and life-management: Possibilities and limits in late adulthood. Ageing and society.

[CR18] Drewelies Johanna, Huxhold Oliver, Gerstorf Denis (2019). The role of historical change for adult development and aging: Towards a theoretical framework about the how and the why. Psychology and Aging.

[CR19] Erikson EH (1950). Childhood and society.

[CR20] Erikson EE, Erikson JM (1997). The life cycle completed.

[CR21] Faber VM (2001). Successful aging in the oldest old: Who can be characterized as successfully aged?. Archives of Internal Medicine.

[CR22] Ferring D, Rosa A, Valsiner J (2018). The experience of aging: Views from without and within. The Cambridge handbook of sociocultural psychology.

[CR23] Finch CE (2010). Evolution of the human lifespan and diseases of aging: Roles of infection, inflammation, and nutrition. Proceedings of National Academy of Sciences of America.

[CR24] Freund, A. M., & Baltes, P. B. (1998). Selection, optimization, and compensation as strategies of life management: Correlations with subjective indicators of successful aging. *Psychology and Aging,**13*, 531–543. 10.1037/00223514.82.4.642. 10.1037//0882-7974.13.4.531 9883454

[CR25] Freund AM, Napolitano CM, Knecht M, Pachana N (2017). Life management through selection, optimization, and compensation. Encyclopedia of geropsychology.

[CR26] Gaugler, J. E. (2015). Innovations in long-term care. In L. K. George & K. F. Ferraro (Eds.), *Handbook of aging and the social sciences* (8th ed., pp. 419–438). London: Academic Press.

[CR27] Gerstorf D, Ram N, Estabrook R, Schupp J, Wagner GG, Lindenberger U (2008). Life satisfaction shows terminal decline in old age: Longitudinal evidence from the German socio-economic panel study (SOEP). Developmental Psychology.

[CR28] Gerstorf D, Ram N, Goebel J, Schupp J, Lindenberger U, Wagner GG (2010). Where people live and die makes a difference: Individual and geographic disparities in well-being progression at the end of life. Psychology and Aging.

[CR29] Gerstorf D, Hülür G, Drewelies J, Eibich P, Duezel S, Demuth I, Ghisletta P, Steinhagen-Thiessen E, Wagner GG, Lindenberger U (2015). Secular changes in late-life cognition and well-being: Towards a long bright future with a short brisk ending?. Psychology and Aging.

[CR30] Gignac M. A. M., Cott C., Badley E. M. (2000). Adaptation to Chronic Illness and Disability and Its Relationship to Perceptions of Independence and Dependence. The Journals of Gerontology Series B: Psychological Sciences and Social Sciences.

[CR31] Gilleard C, Higgs P (2010). Aging without agency: Theorizing the fourth age. Aging & Mental Health.

[CR32] Gilleard C, Higgs P (2011). Ageing abjection and embodiment in the fourth age. Journal of Aging Studies.

[CR33] Hank K (2011). How ‘successful’ do older Europeans age? Findings from SHARE. The Journals of Gerontology, Series B: Psychological Sciences and Social Sciences.

[CR34] Harper S (2014). Economic and social implications of aging societies. Science.

[CR35] Havighurst RJ (1961). Successful aging. The Gerontologist.

[CR36] Havighurst RJ, Albrecht R (1953). Older people.

[CR37] Jopp DS, Wozniak D, Damarin AK, De Feo M, Jung S, Jeswani S (2015). How could lay perspectives on successful aging complement scientific theory? Findings from a U.S. and a German life-span sample. Gerontologist.

[CR38] Kirkwood TBL (2017). Why and how we are living longer?. Experimental Physiology.

[CR39] Kunzmann U, Little TD, Smith J (2000). Is age-related stability of subjective well-being a paradox? Cross-sectional and longitudinal evidence from the Berlin aging study. Psychology and Aging.

[CR40] Laslett P (1989). A fresh map of life: The emergence of the third age.

[CR41] Lindenberger U, Reischies F, Baltes PB, Mayer KU (1999). Limits and potential of intellectual functioning in old age. The Berlin aging study: Aging from 70 to 100.

[CR42] Maddox GL (1987). Aging differently. The Gerontologist.

[CR43] McLaughlin SJ, Connell CM, Heeringa SG, Li LW, Roberts JS (2010). Successful aging in the United States: Prevalence estimates from a national sample of older adults. The Journals of Gerontology, Series B: Psychological Sciences and Social Sciences.

[CR44] Mund Marcus, Freuding Maren M., Möbius Kathrin, Horn Nicole, Neyer Franz J. (2019). The Stability and Change of Loneliness Across the Life Span: A Meta-Analysis of Longitudinal Studies. Personality and Social Psychology Review.

[CR45] Nelson EA, Dannefer D (1992). Aged heterogeneity: Fact or fiction? The fate of diversity in gerontological research. The Gerontologist.

[CR46] Neugarten BL, Havighurst RJ, Tobin SS (1961). The measurement of life satisfaction. Journal of Gerontology.

[CR47] Our World in Data (2019) https://ourworldindata.org/life-expectancy . Download on October 27, 2019.

[CR48] Pressey SL, Simcoe E (1950). Case study comparisons of successful and problem old people. Journal of Gerontology.

[CR49] Robine J-M, Allard M, Herrmann FR, Jeune B (2019). The real facts supporting Jeanne Calment as the oldest ever human. Journal of Gerontology: Medical Sciences.

[CR50] Rosenmayr, L. (1983) *Die späte Freiheit. Das Alter – ein Stück bewusst gelebten Lebens* [the late freedom. Old age – A piece of consciously lived life]. Berlin: Siedler.

[CR51] Rowe JW, Kahn RL (1987). Human aging: Usual and successful. Science.

[CR52] Rowe JW, Kahn RL (1997). Successful aging. The Gerontologist.

[CR53] Rowe JW, Kahn RL (2015). Successful aging 2.0: Conceptual expansions for the 21st century. *The Journals of Gerontology*. Series B: Psychological Sciences and Social Sciences.

[CR54] Ryff CD (1989). Beyond Ponce de Leon and life satisfaction: New directions in quest of successful ageing. International Journal of Behavioral Development.

[CR55] Ryff CD, Keyes CLM (1995). The structure of psychological well-being revisited. Journal of Personality and Social Psychology.

[CR56] Salomon JA, Wang H, Freeman MK, Vos T, Flaxman AD, Lopez AD, Murray CJ (2012). Healthy life expectancy for 187 countries, 1990–2010: A systematic analysis for the global burden disease study 2010. The Lancet.

[CR57] Schaie KW (2013). Developmental influences on adult intelligence: The Seattle longitudinal study.

[CR58] Smith J, Baltes PB, Baltes PB, Mayer KU (1999). Trends and profiles of psychological functioning in very old age. The Berlin aging study. Aging from 70 to 100.

[CR59] Tesch-Römer, C. (in press). Successful aging. In D. Gu & M. Dupre (Eds.), *Encyclopedia of gerontology and population aging*. New York: Springer.

[CR60] Tesch-Römer C, Wahl H-W (2017). Successful aging and aging with care needs: Arguments for a comprehensive concept of successful aging. The Journals of Gerontology, Series B: Social Sciences.

[CR61] Tornstam L (1989). Gero-transcendence: A reformulation of the disengagement theory. Aging.

[CR62] Valsiner J (1994). Irreversibility of time and the construction of historical developmental psychology. Mind, Culture, and Activity.

[CR63] Vaupel J. W., Zhang Z., van Raalte A. A. (2011). Life expectancy and disparity: an international comparison of life table data. BMJ Open.

[CR64] Vogel N, Schilling OK, Wahl H-W, Beekman ATF, Penninx BWJZH (2013). Time-to-death-related change in positive and negative affect among older adults approachung the end of life. Psychology and Aging.

[CR65] Wahl H-W, Gerstorf D (2018). A conceptual framework for studying COntext dynamics in aging (CODA). Developmental Review.

[CR66] Wahl, H.-W., & Schilling, O. K. (2018). Hohes Alter [Advanced old age]. In W. Schneider & U. Lindenberger (Oerter/Montada), *Entwicklungspsychologie* [Developmental psychology] (8th ed., pp. 319-344). Weinheim, Germany: Beltz Verlag.

[CR67] Wahl, H.-W., & Tesch-Römer, C. (2018). Positive aging and concepts of care: Need for bridge-building instead of separation. In T. Boll, D. Ferring, & J. Valsiner (Eds.), *Cultures of care in aging (pp. 57–82)*. Information Age Publishing.

[CR68] Westerhof GJ, Miche M, Brothers AF, Barrett AE, Diehl M, Montepare JM, Wahl H-W, Wurm S (2014). The influence of subjective aging on health and longevity: A meta-analysis of longitudinal data. Psychology and Aging.

[CR69] Wettstein M, Schilling O, Wahl H-W (2016). Still feeling healthy after all these years: The paradox of subjective stability versus objective decline in very old adults’ health and functioning across five years. Psychology and Aging.

[CR70] World Health Organzation (2019). Healthy aging. https://www.who.int/ageing/healthy-ageing/en/ . Download on November 4, 2019.

[CR71] Wrzus C, Hänel M, Wagner J, Neyer FJ (2013). Social network changes and life events across the life span: A meta-analysis. Psychological Bulletin.

